# *Reg* Gene Expression in Periosteum after Fracture and Its In Vitro Induction Triggered by IL-6

**DOI:** 10.3390/ijms18112257

**Published:** 2017-10-27

**Authors:** Yasuaki Tohma, Yoshiko Dohi, Ryogo Shobatake, Tomoko Uchiyama, Maiko Takeda, Shin Takasawa, Yasuhito Tanaka, Hajime Ohgushi

**Affiliations:** 1 Department of Orthopedic Surgery, Nara City Hospital, 1-50-1 Higashikidera-cho, Nara, Nara 630-8305, Japan; yatohma@naramed-u.ac.jp; 2 Department of Orthopedic Surgery, Nara Medical University, 840 Shijo-cho, Kashihara, Nara 634-8522, Japan; yatanaka@naramed-u.ac.jp; 3 Department of Biochemistry, Nara Medical University, 840 Shijo-cho, Kashihara, Nara 634-8521, Japan; yoshiko-dohi@aroma.ocn.ne.jp (Y.D.); rshobatake@naramed-u.ac.jp (R.S.); uchiyama0403@naramed-u.ac.jp (T.U.); maikot@naramed-u.ac.jp (M.T.); hajime-ohgushi@aist.go.jp (H.O.); 4 Department of Diagnostic Pathology, Nara Medical University, 840 Shijo-cho, Kashihara, Nara 634-8522, Japan; 5 Department of Laboratory Medicine and Pathology, National Hospital Organization Kinki-chuo Chest Medical Center, 1180 Nagasone-cho, Kita-ku, Sakai, Osaka 591-8025, Japan; 6 Department of Orthopedic Surgery, Ookuma Hospital, 2-17-13 Kuise-honmachi, Amagasaki, Hyogo 660-0814, Japan

**Keywords:** bone regeneration, fracture, *Reg* (regenerating gene), IL-6, periosteum, *Bim*

## Abstract

The periosteum is a thin membrane that surrounds the outer surface of bones and participates in fracture healing. However, the molecular signals that trigger/initiate the periosteal reaction are not well established. We fractured the rat femoral bone at the diaphysis and fixed it with an intramedullary inserted wire, and the expression of regenerating gene (*Reg*) *I*, which encodes a tissue regeneration/growth factor, was analyzed. Neither bone/marrow nor muscle showed *Reg* *I* gene expression before or after the fracture. By contrast, the periosteum showed an elevated expression after the fracture, thereby confirming the localization of *Reg I* expression exclusively in the periosteum around the fractured areas. Expression of the *Reg* family increased after the fracture, followed by a decrease to basal levels by six weeks, when the fracture had almost healed. In vitro cultures of periosteal cells showed no *Reg I* expression, but the addition of IL-6 significantly induced *Reg I* gene expression. The addition of IL-6 also increased the cell number and reduced pro-apoptotic gene expression of *Bim*. The increased cell proliferation and reduction in *Bim* gene expression were abolished by transfection with *Reg I* siRNA, indicating that these IL-6-dependent effects require the *Reg I* gene expression. These results indicate the involvement of the IL-6/Reg pathway in the osteogenic response of the periosteum, which leads to fracture repair.

## 1. Introduction

Bone tissue can be regenerated through osteogenic differentiation of undifferentiated cells into bone-forming osteoblasts. This regeneration is frequently seen during fracture repair, which produces new bone without scarring. Two types of bone formation processes are known: (1) endochondral bone formation, in which a cartilaginous mass appears, followed by vascular invasion/new bone formation; and (2) intramembranous bone formation, in which new bone forms directly through cascades of osteogenic differentiation without cartilaginous mass formation [1].

Fractures often occur in the mid-shaft (diaphysis) regions of long bones. The diaphysis consists of cortical bone and marrow/fat tissues, and its outer surface is covered with a membrane called the periosteum [2]. Shapiro [1] effectively summarizes the fracture repair process. At the periphery of the fracture site (vascular rich areas), the periosteum, which is elevated from the cortex surface, lays down intramembranous woven bone against the surface. Closer to the fracture site, the tissue damage interrupts the blood supply and leads to the formation of cartilaginous masses both outside and within the cortices. These areas eventually show endochondral bone formation. The chondrocytes within the fracture areas are reported to derive predominantly from the periosteum [3]. When there is little motion around the fractured ends due to stable fracture fixation, woven bone is first produced by the periosteal tissue, followed by lamellar bone formation without the intervention of cartilage tissue formation. Thus, the periosteum is essential for new bone formation during the fracture repair in the cascades responsible for both endochondral and intramembranous bone formation. Many regulatory signals, such as Wnt, Indian hedgehog, bone morphogenetic proteins, Smads 1–8, transforming growth factor (TGF)-β superfamily, transcription factor Runt-related transcription factor 2 (Runx2), etc., reportedly affect bone formation [4,5]. However, little is known about the nature of the molecules that trigger the periosteal reaction that promotes fracture repair.

Many approaches have been reported for the investigation of various tissue regenerations, and we have conducted extensive research that focuses on pancreatic β cell regeneration [6,7,8]. We screened a cDNA library from regenerating islets to isolate a novel gene *Reg* (Regenerating Gene) expressed in the islets [9]. This gene was subsequently found in human tissues [9,10]. The *Reg* gene encodes a C-type lectin, and structurally related molecules (*Reg* family genes) have been identified. These genes are expressed in regenerating pancreatic β cells as well as in the other tissues, such as liver, stomach, intestine etc., and are thought to be involved in cell proliferation and differentiation in these tissues [7,8,9,10,11,12,13]. We also reported *Reg* gene expression in regenerating nerve and skeletal muscle tissues [14,15], and we found a positive relation between this gene expression and survival (regenerating capability) of vascular grafts [16]. Thus, *Reg* gene expression may be crucial for the regeneration of several tissues.

We demonstrated that Reg protein induces β cell replication during pancreatic regeneration via the Reg receptor. Administration of interleukin-6 (IL-6) together with dexamethasone (Dex) induced the formation of an active transcriptional complex for *Reg* and finally triggered the *Reg* gene expression in β cells. We recently found that IL-6 and Dex induced *REG Iα* and *REG Iβ* expression in human β cells [17]. These findings indicate that *Reg* gene expression clearly has an important role in tissue regeneration; however, there are no reports that study how *Reg* gene expression relates to bone tissue regeneration. Therefore, we investigated the *Reg* gene expression during the cascade of rat femoral bone fracture repair as well as the expression in periosteum-derived cell cultures. Here, we report a high level of *Reg* gene expression in periosteal areas after a fracture. We also show that *Reg* gene expression is drastically activated by the addition of IL-6 to the medium of periosteum-derived cell cultures. Furthermore, the IL-6 addition down-regulated *Bim* (Bcl-2-like protein 11) gene expression. This study is the first to show evidence for the involvement of *Reg* gene expression in fracture repair (i.e., bone tissue regeneration). The study also discusses the possible role of apoptosis/anti-apoptosis cascades in the regeneration.

## 2. Results

### 2.1. Regenerating Gene (Reg) I Gene Expression in the Periosteum of Fractured Bone

**Figure 1:**
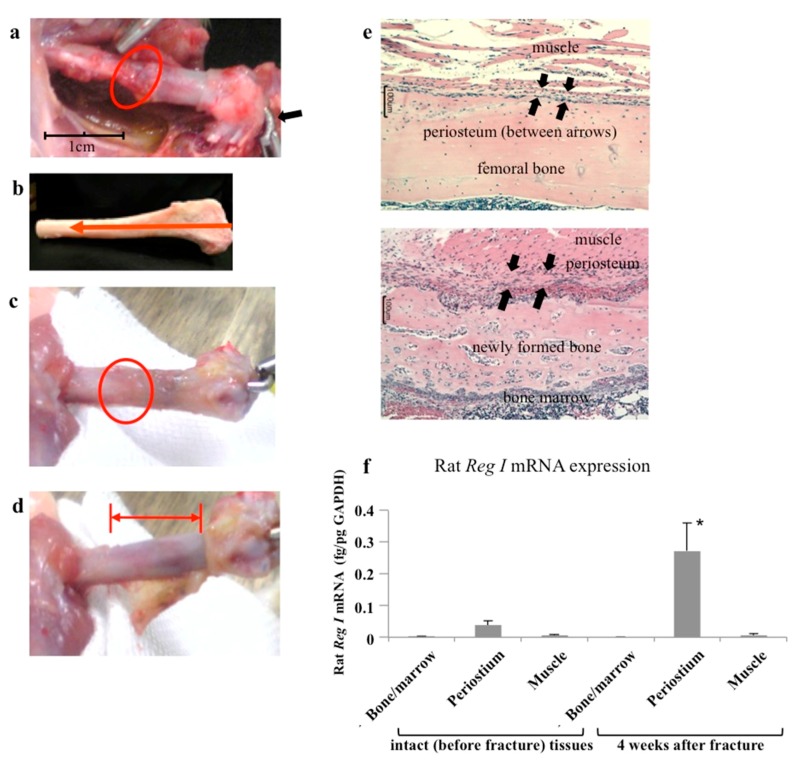
In vivo model of a rat bone fracture. (**a**) A fracture was created at the mid-shaft of the rat femoral bone (red circle). The figure shows fracture fixation with a Kirschner wire (K-wire). The distal edge of the wire is bent (arrow); (**b**) Image and direction of the wire insertion. The direction is from the distal femora bone to the pelvic bone; (**c**) After 4 weeks of fixation, good bone union (red circle) is evident; (**d**) The periosteum covering the fractured bone was removed/harvested. The red bar area indicates bare bone; (**e**) The upper figure shows the histological section of the mid-shaft of an intact rat femoral (no fracture) bone. A thin layer of the periosteum (arrows) is evident between the muscle layer and bone surface. The lower figure shows the fracture area after four weeks. A thick periosteal layer surrounds newly formed bone; (**f**) The expression of *Reg I* in the periosteum. The mRNA levels of rat *Reg I* in bone marrow, periosteum, and muscle tissues before (intact) and four weeks after fracture (*n* = 6). *Reg I* mRNA levels were measured by real-time reverse transcription-polymerase chain reaction (RT-PCR) using *glyceraldehyde-3 phosphate dehydrogenase* (*GAPDH*) as an endogenous control (fg/pg GAPDH). The data are indicated by mean ± SE. * *p* < 0.05.

We made a fracture at the mid-shaft of a rat femoral bone and then stably fixed the fracture site with a wire inserted in the intramedullary region. Bone union was seen after about four weeks and was almost complete by six weeks after fixation (Figure 1a–e). We analyzed *Reg I* gene expression in tissues around the fracture areas by harvesting muscle surrounding the femoral bone, the thin layer of periosteum covering the femoral bone, and the remaining femoral bone that contained bone marrow. The periosteum was easily identified and harvested from the bone. As shown in Figure 1f, neither bone marrow nor muscle showed *Reg I* gene expression before or after the fracture. By contrast, the periosteum showed basal expression of the *Reg I* before the fracture and prominently elevated expression after the fracture. Thus, the *Reg I* gene expression was exclusively localized in the periosteum around the fractured areas.

### 2.2. Expression Profile of Reg Family Genes in the Periosteum of Fractured Bone

**Figure 2:**
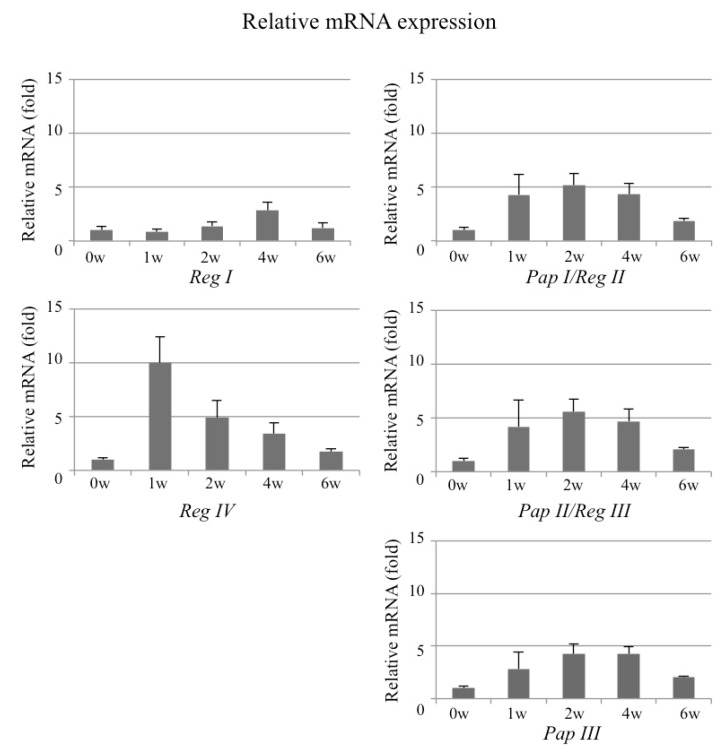
The expression of *Reg* family genes in the periosteum. The mRNA levels of rat Reg family genes (*Reg I*, *Reg IV*, *Pap I*/*Reg II*, *Pap II*/*Reg III*, and *Pap III*) in the periosteum before (0) and 1, 2, 4, and 6 weeks after the fracture (*N* = 4 to 8). The data are indicated by mean ± SD. The mRNA levels before fracture (0 w) are set at 1. The level of *Reg I* mRNA at 4 weeks fracture was significantly increased (*p* = 0.042 vs. 0 week). The levels of *Pap I*/*Reg II* mRNA at 1, 2, 4, and 6 weeks after fracture were significantly increased (*p* = 0.0418, 0.0025, 0.0047, and 0.0254, respectively). The levels of *Pap II*/*Reg III* mRNA at 2, 4, and 6 weeks after fracture were significantly increased (*p* = 0.0080, 0.0063, and 0.0150, respectively). The *Pap III* mRNA levels at 2, 4, and 6 weeks after fracture were increased (*p* = 0.0113, 0.0005, and 0.0045, respectively). The *Reg IV* mRNA levels at 1, 2, and 4 weeks after fracture were increased (*p* = 0.0006, 0.0403, and 0.0267, respectively). w = week.

We also studied expression of all the rat *Reg* family genes (*Reg I*, *Pap I*/*Reg II*, *Pap II*/*Reg III*, *Pap III*, and *Reg IV*) in the periosteum at multiple time points (before and at 1, 2, 4, and 6 weeks after the fracture). As shown in Figure 2, *Reg I* gene expression gradually increased after the fracture and peaked at 4 weeks, when bone union was also detected. A comparison with this *Reg I* gene expression revealed that *Reg IV* gene expression appeared much earlier, with expression noticeable even one week after the fracture. *Pap I*/*Reg II, Pap II*/*Reg III*, and *Pap III* gene expressions showed similar patterns, with peaks around two weeks after the fracture.

The in vivo studies showed baseline expression of all the *Reg* family genes before the fracture and increased expressions following the fracture. The expression of the *Reg* family genes gradually increased and concomitantly decreased with the fracture repair.

### 2.3. The mRNA Levels of Reg I and Its Receptor (Extl3) in Cultured Periosteum-Derived Mesenchymal Stem Cells

**Figure 3:**
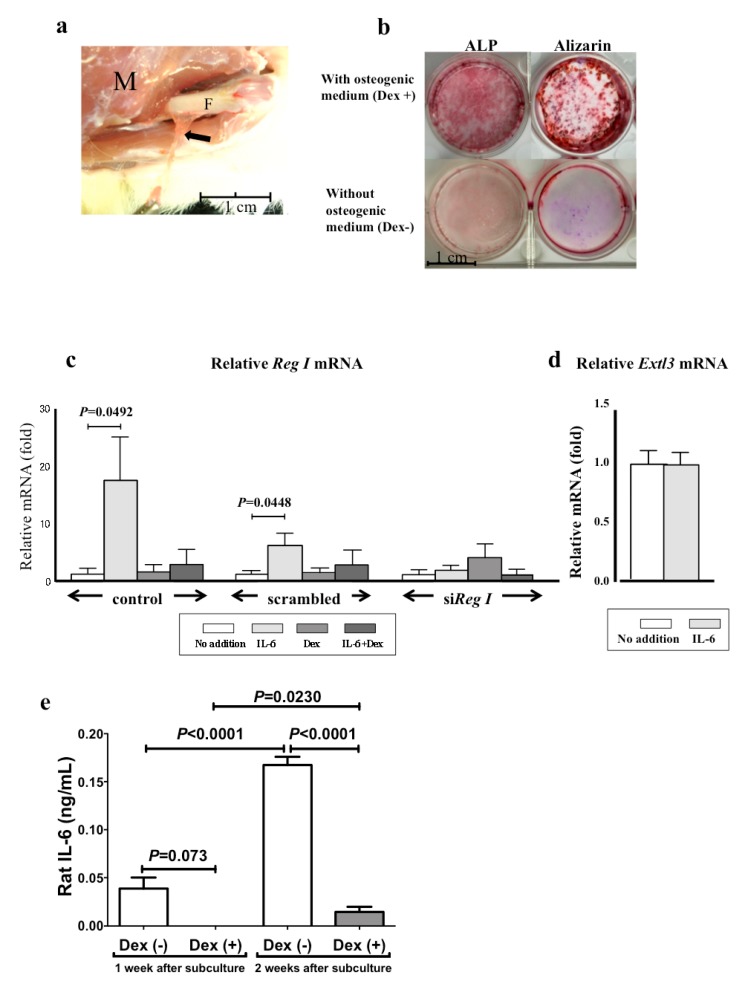
(**a**) Harvesting the periosteum from the femoral bone shaft. The periosteum is easily separated and peeled from the shaft. M: surrounding muscle tissue. F: shaft of the femoral bone. Arrows: periosteum; (**b**) Alkaline phosphatase (ALP) and Alizarin Red S staining of periosteum-derived mesenchymal stem cells (PMSCs) sub-cultured with (upper figures) or without (lower figures) osteogenic medium supplemented with dexamethasone (Dex), ascorbic acid, and β-glycerophosphate for two weeks; (**c**) The mRNA levels of *Reg I* in the culture of PMSCs. Rat PMSCs were transfected with siRNA for *Reg I*, *scrambled* RNA, or no addition (control). After siRNA introduction, IL-6 (20 ng/mL), Dex (100 nM), or IL-6+Dex was added to the PMSC culture medium and the cells were incubated for 24 h. Cells were harvested for real-time RT-PCR for *Reg I* mRNA; (**d**) The mRNA levels of *Extl3* in the culture of rat PMSCs. RNA was prepared from rat PMSCs stimulated with or without IL-6 (20 ng/mL) for 24 h, and Reg receptor (*Extl3*) mRNA was measured by real-time RT-PCR. The data are relative values compared with the mRNA levels of “No addition” and are reported as mean ± SE (*N* = 4); (**e**) Concentration of IL-6 in the medium of the PMSCs sub-cultured with (Dex (+)) or without (Dex (-)) osteogenic supplements for one and two weeks.

We harvested the periosteum from the mid-shaft of intact rat femoral bones (Figure 3a), treated it with collagenase, and cultured the released cells in a basal culture medium. These periosteum-derived cells were well attached to the culture dish and assumed a fibroblastic cell morphology, and they became nearly confluent after two weeks [18]. The culture was followed by subculture in 12-well plates in the same medium with or without osteogenic supplements (Dex, ascorbic acid, and β-glycerophosphate). As shown in Figure 3b, high alkaline phosphatase (ALP) and mineral stain were detected in the subcultures with osteogenic media. Quantitative measurements of the ALP activity of the subculture with osteogenic medium was 0.66 ± 0.10 μmol/30 min/well after seven days and 1.67 ± 0.26 μmol/30 min/well after 14 days. Thus, the cultured periosteal cells were mesenchymal stem cell types that showed osteogenic differentiation capability, and these cells are referred to as periosteum-derived mesenchymal stem cells (PMSCs) in this paper.

We analyzed the *Reg I* gene expression using PMSCs cultured for nine days in the basal medium with or without IL-6 and Dex. We previously reported that the *Reg* gene is activated by IL-6 and Dex and that a combination of IL-6 and Dex synergistically activates this gene expression in pancreatic β cells [7,8,19,20]. As shown in Figure 3c, no *Reg I* expression was detected in the PMSCs, but the addition of IL-6 to the culture medium significantly induced *Reg I* gene expression. By contrast, Dex did not increase *Reg I* gene expression, but it inhibited the IL-6-induced *Reg I* expression (left panel). Transfection with *Reg I* small interfering RNA (siRNA), but not with scrambled siRNA, completely blocked IL-6-induced expression of *Reg I* (middle and right panels). 

The Reg protein is a secretory protein and binds to the Reg protein receptor (*Extl3*) [21]. Therefore, we also analyzed *Extl3* mRNA expression in PMSCs. The level of *Extl3* mRNA in PMSCs was not changed by IL-6 treatment (Figure 3d), suggesting that the effect of IL-6 is due to the up-regulation of *Reg I* mRNA rather than the up-regulation of its receptor. 

We also measured IL-6 protein in the PMSCs culture medium during osteogenic differentiation (one- and two-weeks subculture). As seen in Figure 3e, in contrast to in the non-osteogenic medium lacking Dex, the levels of IL-6 protein in the osteogenic culture medium containing Dex were extremely low, suggesting that *Reg I* mRNA is increased by IL-6 and that Dex decreased *Reg I* expression via the decreased expression of IL-6 in the culture medium.

### 2.4. Proliferation of Periosteum-Derived Mesenchymal Stem Cells PMSCs by Interleukin-6 (IL-6) via Reg I

**Figure 4:**
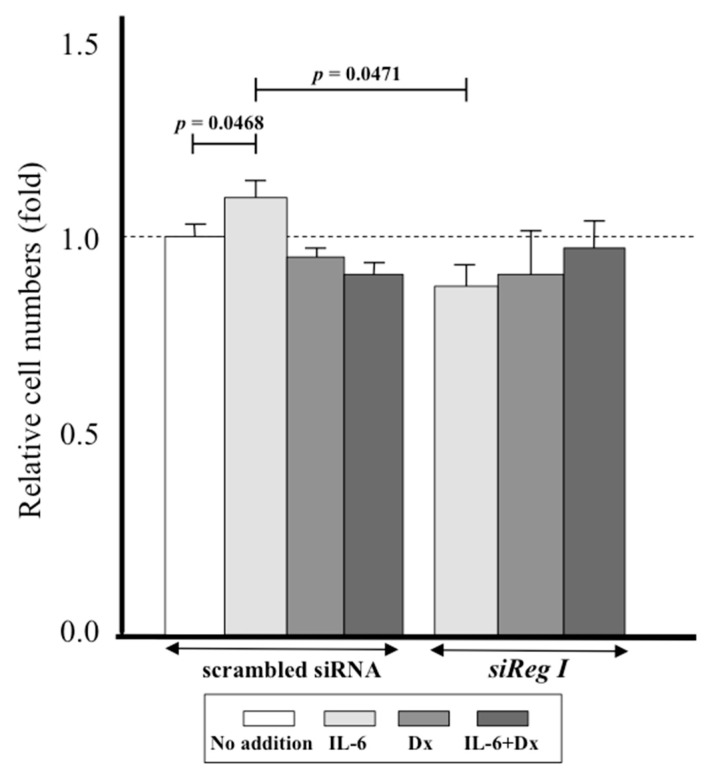
Effects of transfection with siRNA for *Reg I* on cell numbers of PMSCs. The WST-8 assay was performed using siRNA-transfected PMSCs (scrambled siRNA or *siReg I*). These PMSCs were stimulated with IL-6, Dex, and IL-6+Dex or without stimulants (No addition), and the relative cell numbers were measured by cleavage of WST-8. The data are relative values compared with the absorbance of cells with “No addition” and indicated by mean ± SE (*n* = 4).

We then examined the effect of IL-6 on cell proliferation using the 2-(2-methoxy-4-nitrophenyl)-3-(4-nitrophenyl)-5-(2,4-disulfophenyl)-2*H*-tetrazoliummonosodium salt (WST-8) assay. The addition of IL-6 to the culture medium of the PMSCs increased the cell numbers, and this effect was abolished by transfection of PMSCs with *Reg I* siRNA (Figure 4). In contrast, the addition of Dex, either singly or in combination with IL-6, had no effects on the proliferation of PMSCs. Relative cell numbers of IL-6+Dex was significantly reduced (*p* = 0.001 vs. IL-6 alone).

### 2.5. Down–Regulation of Bim, an Apoptosis-Related Gene, in PMSC by IL-6

**Figure 5:**
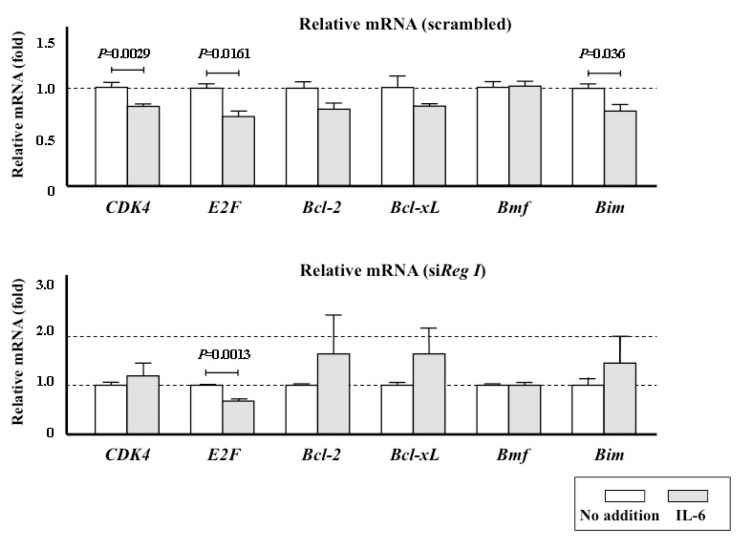
The expression of cell cycle-related (*CDK4* and *E2F*), anti-apoptosis-related (*Bcl-2* and *Bcl-xL*), and apoptosis-related (*Bmf* and *Bim*) genes in PMSCs. Scrambled RNA (**upper** panel) or siRNA for rat *Reg I* (**lower** panel) was introduced into the cells. The measurements were done without the addition of IL-6 to the medium (No addition) and with the addition of IL-6. The data are relative values compared with the mRNA levels from cells with “No addition” and are reported as mean ± SE (*n* = 4).

We also investigated the intracellular mechanism underlying the enhancement of PMSC proliferation by the IL-6/Reg I pathway by analyzing the expression of cyclin-dependent kinase (*CDK4*) and *E2F*. IL-6 stimulation did not increase but rather decreased the mRNA levels of either of these two positive cell cycle regulators (Figure 5). Cell proliferation is a key process in regeneration of many tissues, and apoptotic stimuli affect the cell numbers. We previously reported that the *Reg I* protein has both trophic and anti-apoptotic effects [22,23,24]. We therefore examined the expression of several apoptosis/anti-apoptosis related genes: *Bcl-2* (B cell lymphoma 2), *Bcl-xL* (B-cell lymphoma-extra large), *Bmf* (Bcl-2-modifying factor), and *Bim*. We found that *Bcl-2*, *Bcl-xL*, and *Bmf* mRNA expressions were unchanged following IL-6 stimulation (*Bcl-2* and *Bcl-xL* showed decreased tendency by IL-6 stimulation although it was not statistically significant.). By contrast, IL-6-stimulated PMSCs showed down-regulation of *Bim*, which is an apoptosis-related gene required for apoptosis in a broad range of cell types [25] (Figure 5). This down-regulation of *Bim* was abolished by transfection with *Reg I* siRNA.

## 3. Discussion

Bone formation following heterotopic transplantation of the periosteum, with or without a scaffold, has been reported, supporting the inherent osteogenic capability of this tissue [26,27,28]. The fracture of a rat femoral bone in the present study was repaired after about 4–6 weeks by extensive new bone formation that united the fracture ends. This new bone formation was exclusively derived from the periosteal reaction (Figure 1); thus, this rat model is suitable for investigating the role of the periosteum in fracture repair.

We previously reported the importance of the *Reg* family gene expressions in regenerating tissues, especially during pancreatic tissue regeneration [6,7,8,12]. The *Reg* genes are not expressed under non-regenerating physiological conditions but are expressed during the regeneration of β cells. As was noted for pancreatic tissue regeneration, *Reg* gene expression was difficult to detect in normal (before fracture) periosteum tissue, but expression was clearly evident during the fracture repair process. No previous studies have reported *Reg* family expressions in bone tissue regeneration (fracture repair), but the findings presented here clearly show this expression after the fracture (Figure 1f and Figure 2). The earlier expression of *Reg IV* [29,30,31] compared with *Reg I* may indicate the primary role of *Reg IV* in bone tissue regeneration.

Based on these in vivo findings, we further focused on the study of *Reg* using in vitro culture of periosteum-derived cells. The osteogenic capability of cultured cells derived from the periosteum has been reported previously [32,33]. These cells also differentiate into multiple mesenchymal lineages [33]. Traditionally, mesenchymal stem cells (MSCs) reside in bone marrow and we have previously reported the in vitro osteogenic differentiation of the bone marrow-derived MSCs in osteogenic medium containing Dex, ascorbic acid, and β-glycerophosphate [34]. Periosteum-derived cells cultured under the same conditions showed clear osteogenic differentiation capability, as indicated by a high ALP activity and mineral deposition (Figure 3b). Our previous study indicated that these culture conditions generate cells positive for CD29 and CD90 cell surface antigens but negative for CD45 [18]. Based on these previous findings and the observed osteogenic differentiation capability of the cultured periosteum cells, we adopted the term PMSCs for the cultured cells described here.

In vivo experiments showed that the periosteum expressed the *Reg I* gene at a basal level before the fracture (Figure 1f). Likewise, PMSCs from intact bone did not show apparent *Reg I* expression (Figure 3c). By contrast, after the fracture, the periosteum showed prominent *Reg I* gene expression (Figure 1f and Figure 2) and the in vitro cultured PMSCs that were cultured in vitro showed a drastically increased gene expression following IL-6 stimulation (Figure 3c). Therefore, both fracturing and IL-6 can apparently trigger gene expression in periosteal cells, suggesting that IL-6 plays a role in fracture repair. In this regard, bone tissue harvested from IL-6 knockout mice showed reduced crystallinity, mineral/matrix ratio, tissue mineral density, and bone volume fraction when compared to wild-type mice [35]. Furthermore, the knockout mice showed impaired fracture healing [35].

Kidd et al., using a stress fracture model, reported that even 4 h after a fracture, a marked (220-fold) increase was observed in expression of the IL-6 gene. They proposed that the early up-regulation of IL-6 and IL-11 demonstrates the central role in initiating signaling events for fractures [36]. Others have also reported that sclerostin, VEGF, TGF-β, COX-2, and IL-6 are early signals that facilitate the formation of periosteal woven bone [37]. Glycoprotein 130 (gp130), a co-receptor subunit for transducing signals in response to IL-6 family cytokines, has also been suggested as a possible contributor to bone formation [38,39]. Overall, the available evidence suggests that IL-6 has an important role in the cascade leading to fracture healing. Furthermore, IL-6 is linked to osteoclastogenesis [35,40] and triggers *Reg* gene expression in the periosteal area. Therefore, activation of the IL-6/*Reg* pathway is a prerequisite for fracture healing.

In cultured pancreatic β cells, the *Reg* gene is synergistically activated by IL-6 and Dex [7,8,17,41,42]. However, as shown in Figure 3c, Dex did not stimulate the gene expression; instead, it inhibited the induction of *Reg I* gene expression by IL-6. Thus, PMSCs and pancreatic β cells exhibited different responses to Dex. Furthermore, in the PMSCs culture, Dex seems to suppress the IL-6 gene expression, as evidenced by the little amount of IL-6 protein in the osteogenic medium that contained Dex compared with the medium that lacked Dex (Figure 3e). As described above, Dex also induces osteogenic differentiation in PMSCs. The Dex-induced MSC differentiation into osteoblasts is reported to occur by activation of the Runx2 expression that is dependent on the four and a half LIM domains protein 2 (FHL2)/β-catenin signaling pathway, which is essential for osteogenic differentiation. FHL2 is upregulated in response to Dex, presumably because Dex binds to a glucocorticoid response element in the promoter of FHL2 [43]. The presence of Dex-dependent FHL2 upregulation may reflect a different response of Dex. We also reported that IL-6 significantly enhanced *REG Iα* promoter activity in human salivary ductal cells and that supplementation with Dex had no additional effect on this activity [44]. Thus, the synergistic effect of Dex on IL-6 induced *Reg* gene expression seems to be cell-type dependent.

IL-6 also increased the cell number of PMSCs in culture, and this effect was not seen following transfection with *Reg I* siRNA (Figure 4). A well-organized balance between cell proliferation and apoptosis is a key for normal tissue homeostasis and an inequitable induction of apoptosis may suppress cell growth. The *Reg* protein was reported to stimulate cell proliferation [12,22,41,45] and to inhibit apoptosis [23,24]. In the present study, we first analyzed the mRNA expression of two typical cell cycle regulators, *CDK4* and *E2F*, but we found no increase in response to IL-6 stimulation (Figure 5), suggesting that the increased proliferation of PMSCs is not mediated by cell cycle progression. However, down-regulation of *CDK4* mRNA in IL-6-stimulated PMSCs was canceled by the introduction of *siReg I* RNA (Figure 5). As down-regulation of *CDK4* could work as an impediment in cell cycle progression, Reg I may remove such an impediment to proliferation. We also test the possibility of the inhibition of apoptosis through the stimulation of the IL-6-dependent *Reg I* expression.

Two pathways can lead to apoptosis: the extrinsic or death receptor pathway and the intrinsic, or mitochondrial, pathway. In the latter pathway, mitochondria release cytochrome c and activate a caspase cascade that results in programmed cell death [46]. The Bcl-2 family, consisting of Bcl-2 and its homologues, regulate this mitochondrial pathway. Many genes have been identified in the Bcl-2 family: some have anti-apoptotic and some have pro-apoptotic functions [47]. As seen in Figure 5, treatment of PMSC cultures with IL-6 did not affect the expressions of the anti-apoptotic family genes but reduced the pro-apoptotic *Bim* gene expression, which is known to induce cell death in multiple cell types [48]. Therefore, *Reg I* gene expression induced by IL-6 in PMSCs (Figure 3c) could be linked to the inhibition of *Bim* expression and the resulting increase in cell proliferation (Figure 4).

Bone formation is closely associated with blood vessel growth (i.e., new capillary formation due to proliferation and differentiation of the endothelial cells), and endothelial growth factor-A (VEGF-A) is known to regulate this differentiation. In this regard, *Bim* appears to be responsible for the apoptotic death of retinal endothelial cells during oxygen-induced ischemic retinopathy, and the lack of *Bim* leads to increased retinal vascular density [49]. *Bim* is also required for the apoptotic death of tumor endothelial cells and for inhibition of tumor growth by VEGF neutralization [50]. These findings may imply an additional role of *Reg* gene expression at fracture sites; namely, the expression may favor repair due to improvement of the proliferation/differentiation of endothelial cells around the fracture site. Further studies are needed to elucidate the function of *Reg* gene expression in fracture repair, especially regarding apoptosis and endothelial cell differentiation.

In conclusion, expression of *Reg* family genes in the rat periosteum was triggered by femoral fracture, and this expression decreased after 4–6 weeks, when the fracture union was complete. Therefore, the expression pattern coincided well with the process of fracture healing. Cultured PMSCs derived from intact femoral bone did not show *Reg I* expression, but this expression was induced by the addition of IL-6 to the culture medium. The addition of IL-6 also stimulated the proliferation of the PMSCs, together with a reduction in the expression of the pro-apoptotic *Bim* gene. These effects of IL-6 were abolished by transfection of PMSCs with *Reg I* siRNA. IL-6 is reported to act as an initial signal for bone fracture; these results may indicate an important role for the IL-6/*Reg* pathway in regulation of the osteogenic capability of the periosteum, which leads to fracture healing.

## 4. Materials and Methods

### 4.1. Animals

Fischer 344 (F344) rats were purchased from Japan SLC, Inc. (Hamamatsu, Japan). Nine-week-old male rats were used for the fracture model. Seven-week-old male rats were used as donors for the in vitro culture experiments. The experimental protocol using these rats was approved by the Animal Care and Use Committee of Nara Medical University (approval number 11936; 9 March 2017).

### 4.2. In Vivo Model of Bone Fracture

Twenty F344 male-specific pathogen-free rats were used in this study. Each cage housed two rats and was equipped with an automatic-water supply apparatus. The rats were anesthetized by intraperitoneal pentobarbital administration (3.5 mg/100 g body weight). A lateral skin incision was performed on the thigh area, and the vastus muscles were carefully divided to expose the femoral bone without injuring the periosteum. Massive injury to soft tissues around the fracture sites was avoided by making a small cut at the mid-shaft of the femoral bone with a mini electric circular saw. The cut did not completely traverse the bone but only scored the bone surface at right angles to the long axis of the bone. The fracture was then made manually. A Kirschner wire (K-wire) with a threaded tip (1.4 mm diameter; DePuy Synthes, Zuchwil, Switzerland) was inserted in an intramedullary position from the femoral condyle to the pelvis using a retrograde method. The distal edge of the wire was bent to prevent migration of the distal femoral fragment. Thus, the fracture was fixed with a K-wire from the knee joint (distal femur) to the pelvic bone. We made bilateral fractures in all rats.

### 4.3. Preparation of Tissue Samples for Gene Expression Analyses after Fracture

Muscle tissue was removed from the fracture area (the mid-shaft of the femoral bone). The bone with periosteum was then harvested from around the fracture. A 10-mm wide section of periosteum from the fracture site was detached using a surgical knife. A 5-mm wide bone fragment, without periosteum, was then harvested from the fracture site using a mini electric circular saw. The harvested bone sample contained marrow tissue. Similar tissue samples from the mid-shaft were harvested by the same methods from a control group without fractures.

### 4.4. In Vitro Culture of Periosteum-Derived Cells

Three F344 male rats were used for the in vitro culture assay. The periosteum was harvested from both mid-shafts of the intact femoral bones of each rat. The harvested periosteum was treated for 1 h with 10 mL phosphate-buffered saline (PBS) containing 3 mg/mL collagenase (collagenase type X filtered; Wako Pure Chemical Industries, Osaka, Japan) and then filtered through 40-μm cell strainers (Falcon^®^ 40 μcell strainer Cell Strainer 40 μm; Corning, Durham, NC, USA). The filtrates were placed into T-75 culture flasks (Falcon^®^ Flasks 250 mL, Corning) for primary culture for 10 days [18]. Cultures were maintained in a humidified atmosphere of 95% air and 5% CO_2_ at 37 °C. The culture medium was renewed three times per week. The medium was a minimal essential medium (MEM; Nacalai Tesque Inc., Kyoto, Japan) containing 15% fetal bovine serum (FBS; JRH Bioscience, Lenexa, KS, USA) and a mixture of antibiotics (100 U/mL penicillin, 100 μg/mL streptomycin, and 0.25 μg/mL amphotericin B; Nacalai Tesque). After 10 days of primary culture, adherent cells with fibroblastic morphology were released using 0.25% trypsin-ethylenediaminetetraacetic acid (Life Technologies, Inc., Burlington, ON, Canada), centrifuged at 900 rpm for 5 min at room temperature and the supernatant was discarded.

The residue of the primary cultured cells was subcultured in the same medium at 2.0 × 10^4^ cells/well in 24-well plates (Falcon^®^ Multiwell 24 well, Corning) for quantitative real-time RT-PCR and 3.0 × 10^3^ cells/well in 96-well plates (Falcon^®^ 96 well, Corning) for viable cell counting using WST-8 assay. The RT-PCR and WST-8 assay methods are described in Section 4.5 and Section 4.6, respectively. After two days of subculturing, the cells were transfected with siRNAs for rat *Reg I* or with scrambled siRNA, then 20 ng/mL IL-6 and/or 100 nM Dex were added into the medium, and the cells were incubated for another 24 h. The treated cells were then used for the real-time RT-PCR and WST-8 assays. The subculture was also done in 12-well plates in the same medium or in an osteogenic medium supplemented with 10 nM Dex, 0.28 mM ascorbic acid-2-phosphate, and 10 mM β-glycerophosphate. ALP activity measurements and mineral staining were done according to our reported methods [18,34]. The concentration of IL-6 in the medium was measured using a Rat IL-6 Platinum ELISA (enzyme-linked immunosorbent assay) kit (Bender MedSystems GmbH, Vienna, Austria) according to the supplier’s instructions.

The Silencer^®^ Select predesigned siRNAs for rat *Reg I* and the scrambled siRNA were purchased from Life Technologies (Carlsbad, CA, USA)*.* The sense sequence of siRNA for the rat *Reg I* was 5′-GAAAUGGAGAGAUAACAGUtt-3′. The cells were transfected with the siRNAs using Lipofectamine^®^ RNAiMAX Reagent (Life Technologies), as previously described [19,20,21,51,52,53]. Cells were transfected with siRNAs at 5 pmol/well in 24-well plates for real-time PCR and 1 pmol/well in 96-well plates for WST-8 assays. IL-6 (Interleukin-6 Rat recombinant) was purchased from Wako Pure Chemical Industries, and Dex was purchased from Sigma (St. Louis, MO, USA).

### 4.5. Quantitative Real-Time RT-PCR 

Total RNA from in vivo samples was isolated using the Isogen RNA Extraction Kit (Nippon Gene, Toyama, Japan), and total RNA from in vitro cultured PMSCs was isolated using the RNeasy^®^ Plus Mini Kit (Qiagen, Hilden, Germany), as previously described [19,20,44,51,52,53]. The corresponding cDNA was synthesized using total RNA (2–5 μg) as a template and a High Capacity cDNA Reverse Transcription Kit (Applied Biosystems Inc., Foster City, CA, USA) was used to produce a template for real-time PCR. The real-time quantitative PCR was performed using a KAPA SYBR^®^ Fast qPCR kit (KAPA Biosystems, Boston, MA, USA) or TaqMan^®^ Fast Universal PCR Master Mix (Applied Biosystems) using a Thermal Cycler Dice Real Time System (Takara, Otsu, Japan), Applied Biosystems StepOne^TM^, or StepOnePlus^TM^ Real-Time PCR System (Japan Applied Biosystems Inc., Tokyo, Japan) with the appropriate primers. The PCR primers were synthesized by Nippon Gene Research Laboratories (Sendai, Japan), and their sequences are listed in Table 1. The thermal cycling conditions were 3 min at 95 °C for activation of polymerase, followed by 40–45 cycles of 3–10 s at 95 °C for denaturation, 5 s at 60 °C for annealing, and 20 s at 60–72 °C for extension. Target cDNAs were cloned into pBluescript SK(-) plasmid (Stratagene, La Jolla, CA, USA), and sequential 10-fold dilutions from 10^2^–10^7^ copies/μL were prepared. The serial dilutions were run to verify the specificity and to test the sensitivity of the SYBR Green-based real-time RT-PCR. Differences in the efficiency of reverse transcription between the samples were adjusted by normalizing the level of target mRNA to the mRNA level of *GAPDH* or ribosomal protein S15 (*Rps15*).

### 4.6. Measurement of Viable Cell Numbers by Tetrazolium Salt Cleavage

The viable cell numbers were determined using a Cell Counting Kit-8 (WST-8; Dojindo, Mashiki-machi, Japan) according to the previously described method based on tetrazolium reductase activity [19,20,45,53]. Briefly, WST-8 solution was added to cells in 96-well plates (10 μL in 100 μL culture medium), and the cells were incubated for 2 h at 37 °C in a humidified atmosphere of 95% air and 5% CO_2_. After incubation, the optical density of each well was read at 450 nm (reference wave length at 650 nm) using a SpectraMax M2 instrument (Molecular Devices, Sunnyvale, CA, USA).

### 4.7. Data Analysis

Multiple comparisons regarding *Reg* gene expressions in the in vivo model were evaluated by one-way analysis of variance with post-hoc multiple comparisons using the Tukey test. A value of *p* < 0.05 was considered statistically significant. The WST-8 assay results were analyzed using the Mann-Whitney *U*-test. A comparison between the two groups was evaluated by a Student’s *t* test using GraphPad Prism (GraphPad Software, La Jolla, CA, USA).

## Figures and Tables

**Table 1:** Primers used for real-time RT-PCR of rat mRNA(s).

Primer	Sequence
*Reg I* Forward	5′-GGACACTGGGTATCCTAACAATTCC-3′ (M18962)
*Reg I* Reverse	5′-CTCTCCATTTCTTGTATCCTGAGTTTG-3′ (M18962)
*Pap I*/*Reg II* Forward	5′-AAAATACCCTCTGCACGCATTAG-3′ (NM_053289)
*Pap I*/*Reg II* Reverse	5′-GGGCATAGCAGTAGGAGCCATA-3′ (NM_053289)
*Pap II*/*Reg III* Forward	5′-CCAGAAGGCAGTGCCCTCTA-3′ (L10229)
*Pap II*/*Reg III* Reverse	5′-GCAGTAAGAACGATAAGCCTTGGA-3′ (L10229)
*Pap III* Forward	5′-TGTGCCCACTTCACGTATCAG-3′ (L_20869)
*Pap III* Reverse	5′-GGCATAGCAATAGGAGCCATAGG-3′ (L_20869)
*Reg IV* Forward	5′-CTGCTGAGCTGGGTAGCTGGCCC-3′ (AB164049)
*Reg IV* Reverse	5′-TTTATCCTTGGGGTTCATCTCAG-3′ (AB164049)
*Extl3* Forward	5′-CAATCGGTTCTTGCCCTGG-3′ (NM_020097)
*Extl3* Reverse	5′-GGAAGTTCATGGCGATATCC-3′ (NM_020097)
*CDK4* Forward	5′-TTTGATCTCATTGGATTGCC-3′ (NM_053593)
*CDK4* Reverse	5′-AGGTCAGCATTTCCAGCAG-3′ (NM_053593)
*E2F* Forward	5′-TTCTTGGAGCTGCTGAGCC-3′ (NM_001100778)
*E2F* Reverse	5′-TGGTGATGTCATAGATGCG-3′ (NM_001100778)
*Bcl-2* Forward	5′-CGGGAGAACAGGGTATGA-3′ (NM_016993)
*Bcl-2* Reverse	5′-CAGGCTGGAAGGAGAAGAT-3′ (NM_016993)
*Bcl-xL* Forward	5′-TCTAACATCCCAGCTTCAT-3′ (NM_001033672)
*Bcl-xL* Reverse	5′-GCAATCCGACTCACCAATA-3′ (NM_001033672)
*Bmf* Forward	5′-GAGACGCTGTCCTGGAGTCA-3′ (NM_139258)
*Bmf* Reverse	5′-GGCCTTGTCTTCCTGGCTTA-3′ (NM_139258)
*Bim* Forward	5′-GCCAAGCAACCTTCTGATGTA-3′ (NM_171989)
*Bim* Reverse	5′-CAGTGCCTTCTCCAGACCAG-3′ (NM_171989)
*Rig*/*rpS15* Forward	5′-ACGGCAAGACCTTCAACCAG-3′ (NM_001018)
*Rig*/*rpS15* Reverse	5′-ATGGAGAACTCGCCCAGGTAG-3′ (M_001018)
*GAPDH* Forward	5′-AACGACCCCTTCATTGACCTC-3′ (NM_017008)
*GAPDH* Reverse	5′-CCTTGACTGTGCCGTTGAACT-3′ (NM_017008)

## Abbreviations

TGF	Transforming growth factor
Runx2	Runt-related transcription factor 2
*Reg*	Regenerating gene
Dex	Dexamethasone
IL-6	Interleukin-6
RT-PCR	Reverse transcription-polymerase chain reaction
Extl3	Reg protein receptor
PMSC	Periosteum-derived mesenchymal stem cell
ALP	Alkaline phosphatase
WST-8	2-(2-Methoxy-4-nitrophenyl)-3-(4-nitrophenyl)-5-(2,4-disulfophenyl)-2*H*-tetrazoliummonosodium salt
CDK4	Cyclin-dependent kinase
Bcl-2	B Cell lymphoma 2
Bcl-xL	B-Cell lymphoma-extra large
*Bmf*	Bcl-2-Modifying factor
*Bim*	Bcl-2-Like protein 11
MSC	Mesenchymal stem cells
FHL2	Four and a half LIM domains protein 2
VEGF	Endothelial growth factor
siRNA	Small interfering RNA

## References

[1] Shapiro F. (2008). Bone development and its relation to fracture repair. The role of mesenchymal osteoblasts and surface osteoblasts. Eur. Cells Mater..

[2] Squier C.A., Ghoneim S., Kremenak C.R. (1990). Ultrastructure of the periosteum from membrane bone. J. Anat..

[3] Colnot C. (2009). Skeletal cell fate decisions within periosteum and bone marrow during bone regeneration. J. Bone Miner. Res..

[4] Rahman M.S., Akhtar N., Jamil H.M., Banik R.S., Asaduzzaman S.N. (2015). TGF-β/BMP signaling and other molecular events: Regulation of osteoblastogenesis and bone formation. Bone Res..

[5] Hayrapetyan A., Jansen J.A., van den Beucken J.J. (2015). Signaling pathways involved in osteogenesis and their application for bone regenerative medicine. Tissue Eng. Part B Rev..

[6] Yonemura Y., Takashima T., Miwa K., Miyazaki I., Yamamoto H., Okamoto H. (1984). Amelioration of diabetes mellitus in partially depancreatized rats by poly(ADP-ribose) synthetase inhibitors. Evidence of islet B-cell regeneration. Diabetes.

[7] Takasawa S., Okamoto H. (2002). Pancreatic β-cell death, regeneration and insulin secretion: Roles of poly(ADP-ribose) polymerase and cyclic ADP-ribose. Int. J. Exp. Diabetes Res..

[8] Okamoto H., Takasawa S. (2002). Recent advances in the Okamoto model: The CD38-cyclic ADP-ribose signal system and the regenerating gene protein (Reg)-Reg receptor system in β-cells. Diabetes.

[9] Terazono K., Yamamoto H., Takasawa S., Shiga K., Yonemura Y., Tochino Y., Okamoto H. (1988). A novel gene activated in regenerating islets. J. Biol. Chem..

[10] Watanabe T., Yonekura H., Terazono K., Yamamoto H., Okamoto H. (1990). Complete nucleotide sequence of human reg gene and its expression in normal and tumoral tissues. The reg protein, pancreatic stone protein, and pancreatic thread protein are one and the same product of the gene. J. Biol. Chem..

[11] Zhang Y.W., Ding L.S., Lai M.D. (2003). Reg gene family and human diseases. World J. Gastroenterol..

[12] Takasawa S. (2016). Regenerating gene (REG) product and its potential clinical usage. Expert Opin. Ther. Targets.

[13] Watanabe T., Yonemura Y., Yonekura H., Suzuki Y., Miyashita H., Sugiyama K., Moriizumi S., Unno M., Tanaka O., Kondo H. (1994). Pancreatic beta-cell replication and amelioration of surgical diabetes by Reg protein. Proc. Natl. Acad. Sci. USA.

[14] Namikawa K., Fukushima M., Murakami K., Suzuki A., Takasawa S., Okamoto H., Kiyama H. (2005). Expression of Reg/PAP family members during motor nerve regeneration in rat. Biochem. Biophys. Res. Commun..

[15] Klasan G.S., Ivanac D., Erzen D.J., Picard A., Takasawa S., Peharec S., Arbanas J., Girotto D., Jerkovic R. (2014). Reg 3γ gene expression in regenerating skeletal muscle and corresponding nerve. Muscle Nerve.

[16] Kiji T., Dohi Y., Nishizaki K., Takasawa S., Okamoto H., Nagasaka S., Naito H., Yonemasu K., Taniguchi S. (2003). Enhancement of cell viability in cryopreserved rat vascular grafts by administration of regenerating gene (*REG*) inducers. J. Vasc. Res..

[17] Yamauchi A., Itaya-Hironaka A., Sakuramoto-Tsuchida S., Takeda M., Yoshimoto K., Miyaoka T., Fujimura T., Tsujinaka H., Tsuchida C., Ota H. (2015). Synergistic activations of *REG Iα* and *REG Iβ* promoters by IL-6 and glucocorticoids through JAK/STAT pathway in human pancreatic β cells. J. Diabetes Res..

[18] Hayashi O., Katsube Y., Hirose H., Ohgushi H., Ito H. (2008). Comparison of osteogenic ability of rat mesenchymal stem cells from bone marrow, periosteum, and adipose tissue. Calcif. Tissue Int..

[19] Ota H., Itaya-Hironaka A., Yamauchi A., Sakuramoto-Tsuchida S., Miyaoka T., Fujimura T., Tsujinaka H., Yoshimoto K., Nakagawara K., Tamaki S. (2013). Pancreatic β cell proliferation by intermittent hypoxia via up-regulation of *Reg* family genes and *HGF* gene. Life Sci..

[20] Tsujinaka H., Itaya-Hironaka A., Yamauchi A., Sakuramoto-Tsuchida S., Ota H., Takeda M., Fujimura T., Takasawa S., Ogata N. (2015). Human retinal pigment epithelial cell proliferation by the combined stimulation of hydroquinone and advanced glycation end-products via up-regulation of *VEGF* gene. Biochem. Biophys. Rep..

[21] Kito K., Kagami H., Kobayashi C., Ueda M., Terasaki H. (2005). Effects of cryopreservation on histology and viability of cultured corneal epithelial cell sheets in rabbit. Cornea.

[22] Kobayashi S., Akiyama T., Nata K., Abe M., Tajima M., Shervani N.J., Unno M., Matsuno S., Sasaki H., Takasawa S. (2000). Identification of a receptor for Reg (regenerating gene) protein, a pancreatic β-cell regeneration factor. J. Biol. Chem..

[23] Sekikawa A., Fukui H., Fujii S., Takeda J., Nanakin A., Hisatsune H., Seno H., Takasawa S., Okamoto H., Fujimori T. (2005). REG Iα protein may function as a trophic and/or anti-apoptotic factor in the development of gastric cancer. Gastroenterology.

[24] Sekikawa A., Fukui H., Fujii S., Ichikawa K., Tomita S., Imura J., Chiba T., Fujimori T. (2008). REG Iα protein mediates an anti-apoptotic effect of STAT3 signaling in gastric cancer cells. Carcinogenesis.

[25] Youle R.J., Strasser A. (2008). The BCL-2 protein family: Opposing activities that mediate cell death. Nat. Rev. Mol. Cell Biol..

[26] Uddströmer L. (1978). The osteogenic capacity of tubular and membranous bone periosteum. A qualitative and quantitative experimental study in growing rabbits. Scand. J. Plast. Reconstr. Surg..

[27] Ishida H., Tamai S., Yajima H., Inoue K., Ohgushi H., Dohi Y. (1996). Histologic and biochemical analysis of osteogenic capacity of vascularized periosteum. Plast. Reconstr. Surg..

[28] Wiese K.G., Merten H.A. (1993). The role of the periosteum in osteointegration of hydroxyapatite granules. Int. J. Oral Maxillofac. Surg..

[29] Hartupee J.C., Zhang H., Bonaldo M.F., Soares M.B., Dieckgraefe B.K. (2001). Isolation and characterization of a cDNA encoding a novel member of the human regenerating protein family: Reg IV. Biochim. Biophys. Acta.

[30] Kämäräinen M., Heiskala K., Knuutila S., Heiskala M., Winqvist O., Andersson L.C. (2003). RELP, a novel human REG-like protein with up-regulated expression in inflammatory and metaplastic gastrointestinal mucosa. Am. J. Pathol..

[31] Kaprio T., Hagström J., Mustonen H., Koskensalo S., Andersson L.C., Haglund C. (2014). REG4 independently predicts better prognosis in non-mucinous colorectal cancer. PLoS ONE.

[32] Nakahara H., Goldberg V.M., Caplan A.I. (1991). Culture-expanded human periosteal-derived cells exhibit osteochondral potential in vivo. J. Orthop. Res..

[33] Nakahara H., Dennis J.E., Bruder S.P., Haynesworth S.E., Lennon D.P., Caplan A.I. (1991). In vitro differentiation of bone and hypertrophic cartilage from periosteal-derived cells. Exp. Cell Res..

[34] Ohgushi H., Dohi Y., Katuda T., Tamai S., Tabata S., Suwa Y. (1996). In vitro bone formation by rat marrow cell culture. J. Biomed. Mater. Res..

[35] Wallace A., Cooney T.E., Englund R., Lubahn J.D. (2011). Effects of interleukin-6 ablation on fracture healing in mice. J. Orthop. Res..

[36] Kidd L.J., Stephens A.S., Kuliwaba J.S., Fazzalari N.L., Wu A.C., Forwood M.R. (2010). Temporal pattern of gene expression and histology of stress fracture healing. Bone.

[37] Wu A.C., Kidd L.J., Cowling N.R., Kelly W.L., Forwood M.R. (2014). Osteocyte expression of caspase-3, COX-2, IL-6 and sclerostin are spatially and temporally associated following stress fracture initiation. Bonekey Rep..

[38] Johnson R.W., Brennan H.J., Vrahnas C., Poulton I.J., McGregor N.E., Standal T., Walker E.C., Koh T.-T., Nguyen H., Walsh N.C. (2014). The primary function of gp130 signaling in osteoblasts is to maintain bone formation and strength, rather than promote osteoclast formation. J. Bone Miner. Res..

[39] Johnson R.W., McGregor N.E., Brennan H.J., Crimeen-Irwin B., Poulton I.J., Martin T.J., Sims N.A. (2015). 41Glycoprotein130 (Gp130)/interleukin-6 (IL-6) signalling in osteoclasts promotes bone formation in periosteal and trabecular bone. Bone.

[40] Yang X., Ricciardi B.F., Hernadez-Soria A., Shi Y., Pleshko Camacho N., Bostrom M.P. (2007). Callus mineralization and maturation are delayed during fracture healing in interleukin-6 knockout mice. Bone.

[41] Akiyama T., Takasawa S., Nata K., Kobayashi S., Abe M., Shervani N.J., Ikeda T., Nakagawa K., Unno M., Matsuno S. (2001). Activation of *Reg* gene, a gene for insulin-producing β-cell regeneration: Poly(ADP-ribose) polymerase binds *Reg* promoter and regulates the transcription by autopoly(ADP-ribosyl)ation. Proc. Natl. Acad. Sci. USA.

[42] Nakagawa K., Takasawa S., Nata K., Yamauchi A., Itaya-Hironaka A., Ota H., Yoshimoto K., Sakuramoto-Tsuchida S., Miyaoka T., Takeda M. (2013). Prevention of Reg I-induced β-cell apoptosis by IL-6/dexamethasone through activation of *HGF* gene regulation. Biochim. Biophys. Acta.

[43] Langenbach F., Handschel J. (2013). Effects of dexamethasone, ascorbic acid and β-glycerophosphate on the osteogenic differentiation of stem cells in vitro. Stem Cell Res. Ther..

[44] Fujimura T., Fujimoto T., Itaya-Hironaka A., Miyaoka T., Yoshimoto K., Yamauchi A., Sakuramoto-Tsuchida S., Kondo S., Takeda M., Tsujinaka H. (2015). Interleukin-6/STAT pathway is responsible for the induction of gene expression of REG Iα, a new auto-antigen in Sjögren’s syndrome patients, in salivary duct epithelial cells. Biochem. Biophys. Rep..

[45] Takasawa S., Ikeda T., Akiyama T., Nata K., Nakagawa K., Shervani N.J., Noguchi N., Murakami-Kawaguchi S., Yamauchi A., Takahashi I. (2006). Cyclin D1 activation through ATF-2 in Reg-induced pancreatic β-cell regeneration. FEBS Lett..

[46] Happo L., Strasser A., Cory S. (2012). BH3-only proteins in apoptosis at a glance. J. Cell Sci..

[47] Elmore S. (2007). Apoptosis: A review of programmed cell death. Toxicol. Pathol..

[48] Piñon J.D., Labi V., Egle A., Villunger A. (2008). Bim and Bmf in tissue homeostasis and malignant disease. Oncogene.

[49] Wang S., Park S., Fei P., Sorenson C.M. (2011). Bim is responsible for the inherent sensitivity of the developing retinal vasculature to hyperoxia. Dev. Biol..

[50] Naik E., O’Reilly L.A., Asselin-Labat M.L., Merino D., Lin A., Cook M., Coultas L., Bouillet P., Adams J.M., Strasser A. (2011). Destruction of tumor vasculature and abated tumor growth upon VEGF blockade is driven by proapoptotic protein Bim in endothelial cells. J. Exp. Med..

[51] Tsujinaka H., Itaya-Hironaka A., Yamauchi A., Sakuramoto-Tsuchida S., Shobatake R., Makino M., Masuda N., Hirai H., Takasawa S., Ogata N. (2017). Statins decrease vascular epithelial growth factor expression via down-regulation of receptor for advanced glycation end-products. Heliyon.

[52] Uchiyama T., Ota H., Itaya-Hironaka A., Shobatake R., Yamauchi A., Sakuramoto-Tsuchida S., Makino M., Kimura H., Takeda M., Obayashi C. (2017). Up-regulation of *selenoprotein P* and *HIP/PAP* mRNAs in hepatocytes by intermittent hypoxia via down-regulation of miR-203. Biochem. Biophys. Rep..

[53] Tsuchida C., Sakuramoto-Tsuchida S., Takeda M., Itaya-Hironaka A., Yamauchi A., Misu M., Shobatake R., Uchiyama T., Makino M., Pujol-Autonell I. (2017). Expression of *REG* family genes in human inflammatory bowel diseases and its regulation. Biochem. Biophys. Rep..

